# Supplementary material for: MetaRibo-Seq measures translation in microbiomes
Source: Nat Commun. 2020 Jun 29;11:3268. doi: 10.1038/s41467-020-17081-z (PMC7324362; doi:10.1038/s41467-020-17081-z)
Supplement: Supplementary file 10 — Supplementary Data 7 [file 41467_2020_17081_MOESM10_ESM.zip › File2/Confidence_High_Taxonomy/395354_out.krona.html]

Javascript must be enabled to view this page.

members
magnitude
magnitudeUnassigned
count
unassigned
taxon
rank

395354\_out

8

superkingdom
8
2

201174
8
phylum

class
84998
8

order
8
84999

84107
8
family

genus
102106
8

1

SRS098644\_contig\_number\_43710
2292330
species

species
2

SRS014683\_contig\_number\_2173SRS147022\_contig\_number\_1271
1946384

species
2292027
1

SRS014235\_contig\_number\_contig-100\_1077.244666

species
74426
1

SRS023715\_contig\_number\_7188

species
2
2292226

SRS012273\_contig\_number\_contig-100\_1575.252042SRS022071\_contig\_number\_26460

species
1262850
1

SRS015782\_contig\_number\_32557
